# Antibacterial activity against pathogenic *Vibrio* and cytotoxicity on human hepatocyte of nano-silver prepared by polysaccharide-protein complexes

**DOI:** 10.3389/fmicb.2024.1416844

**Published:** 2024-10-30

**Authors:** Peirong He, Wenying Wang, Wenjie Jian

**Affiliations:** ^1^College of Public Health, Fujian Medical University, Fuzhou, China; ^2^Department of Public Health and Medical Technology, Xiamen Medical College, Xiamen, China

**Keywords:** pathogenic *Vibrio*, silver nanoparticles, cytotoxicity, antibacterial activity, polysaccharide-protein complexes, human hepatocyte

## Abstract

Silver nanoparticles (AgNPs) are potential antibacterial agents against pathogenic *Vibrio* bacteria in the field of public health, yet their widespread use is limited by dispersibility and biocompatibility. In a previous study, highly dispersible AgNPs were fabricated using a polysaccharide–protein complex (PSP) obtained from the viscera *of Haliotis discus*. In this study, the antibacterial activity of PSP-AgNPs against pathogenic *Vibrio* and its cytotoxicity for human hepatocytes (LO2) was evaluated. At dosages of 3.125–25.0 μg/mL, PSP-AgNPs demonstrated excellent antibacterial activity against several pathogenic *Vibrio* strains (such as *V. fluvialis*, *V. mimicus*, *V. hollisae, V. vulnificus*, and *V. furnissii*), and no cytotoxicity on LO2 cells. This was evidenced by cellular viability, reactive oxygen species, and antioxidase activities. However, severe cytotoxicity was observed at a PSP-AgNPs concentration of 50.0 μg/mL. Furthermore, intracellular oxidative stress was the predominant mechanism of toxicity induced by PSP-AgNPs. Overall, PSP-AgNPs are highly biocompatible in the range of effective antibacterial dosages, identifying them as promising bactericide candidates in the field of public health.

## 1 Introduction

Diseases caused by infection with pathogenic bacteria of the genus *Vibrio* are common in the field of public health and aquaculture (Brumfield et al., 2021; Fleischmann et al., 2022). These diseases can cause large-scale mortality in all stages of aquatic animal culture and infectious diseases in humans (Brumfield et al., 2021; Neetoo et al., 2022). To control pathogenic *Vibrio* strains, various approaches including antibiotics, probiotics, and plant-based products have been employed in aquaculture (Chandrakala and Parameswari, 2021; Abioye and Okoh, 2018). However, these approaches cannot fully meet practical demand, and have several negative side effects. For example, the overuse of antibiotics leads to the emergence of drug-resistant bacterial strains (Sony et al., 2021; RathnaKumari et al., 2018). Thus, the search for safe and effective antimicrobial agents against pathogenic *Vibrio* strains has become a major research goal worldwide.

Recent developments in silver nanoparticles (AgNPs) have identified these as good alternatives to overcome the above problems, because of their broad-spectrum and efficient efficacy against bacteria, fungi, and antibiotic-resistant pathogens (Serrano-Díaz et al., 2023). Despite their excellent bactericidal effect, the widespread use of AgNPs is commonly limited by their dispersion stability and biological safety resulting from preparation methods (Yang and Wu, 2022). Traditionally, AgNPs are prepared by chemical vapor deposition irradiation or chemical reduction of metal salts with sodium borohydride. These processes result in unsuitable dispensability, harmful by-products, and toxic residues.

To overcome these defects, a suitable polysaccharide-protein (PSP) complex was obtained from the viscera of *Haliotis discus*. PSP has been used for the preparation of AgNPs in a previous study by our team (Sony et al., 2021). The PSP complex plays the role of a reducing and capping agent under a simple redox system of silver nitrate without the addition of a reducing agent. The prepared PSP-AgNPs demonstrated excellent antibacterial activity against *Staphylococcus aureus* (Gram-positive) and *Escherichia coli* (Gram-negative). Additionally, PSP-AgNPs achieved highly stable dispersion even in seawater (Jian et al., 2020).

Overall, to explore the potential application of PSP-AgNPs as an antibacterial agent against pathogenic *Vibrio* strains, it is necessary to measure the antibacterial activity against the main pathogenic *Vibrio* strains and gauge its cytotoxicity. So far, numerous studies demonstrated that one predominant mechanism of toxicity is the intracellular oxidative stress, which is induced by AgNPs in a dose and time-dependent manner (Xue et al., 2018; Komazec et al., 2023). This intracellular oxidative stress leads to cell membrane leakage, mitochondria injury, and subsequent apoptotic cell death (Xue et al., 2016; Salama et al., 2023). Therefore, measuring the oxidative stress is an effective method to determine the toxicity of AgNPs. Oxidative stress can be represented by depletion of glutathione (GSH) as well as induction of reactive oxygen species (ROS), lipid peroxidation, superoxide dismutase (SOD), and catalase (Suliman et al., 2015; Lu et al., 2022). Limited by research capacity, the human hepatocyte cell line (LO2) was chosen as the model system in this study, as an *in vivo* biodistribution study indicated that AgNPs are mainly accumulated in the liver (Xue et al., 2018).

In short, the objective of this study was to evaluate the antibacterial activity against the main pathogenic members of the genus *Vibrio* (*V. fluvialis*, *V. mimicus*, *V. hollisae*, *V. vulnificus*, and *V. furnissii*). Based on the result of antibacterial tests, cytotoxicity and oxidative stress of PSP-AgNPs on LO2 cells was further examined by determining cellular viability, as well as the content of malondialdehyde (MDA), and the activity of lactate dehydrogenase (LDH), glutathione peroxidase (GSH-Px), and SOD.

## 2 Results

### 2.1 Antibacterial activities against pathogenic *Vibrio* strains

The antibacterial activities of PSP-AgNPs against several pathogenic *Vibrio* strains are listed in Table 1. The minimum inhibitory concentration (MIC) and minimum bactericidal concentration (MBC) of PSP-AgNPs against *V. fluvialis* were both 12.5 μg/mL. The corresponding values for PSP-AgNPs against *V. mimicus* were 6.25 and 25.0 μg/mL, respectively. The smallest MIC of 3.125 μg/mL was found in *V. furnissii*, and the smallest MBC value of 12.5 μg/mL was found in *V. fluvialis*, *V. hollisae*, and *V. furnissii*. Thus, it can be concluded that the effective concentration range of PSP-AgNPs against pathogenic *Vibrio* was 3.125–25.0 μg/mL.

**TABLE 1:** Antibacterial activities [minimum inhibitory concentration (MIC) and minimum bactericidal concentration (MBC)] of polysaccharide–protein complex silver nanoparticles (PSP-AgNPs) against various pathogenic *Vibrio* strains.

Bacteria of interest	MIC (μ g/mL)	MBC (μ g/mL)
*Vibrio fluvialis*	12.5	12.5
*Vibrio mimicus*	6.25	25.0
*Vibrio hollisae*	6.25	12.5
*Vibrio vulnificus*	12.5	25.0
*Vibrio furnissii*	3.125	12.5

### 2.2 Cytotoxicity on LO2 cells

**FIGURE 1:**
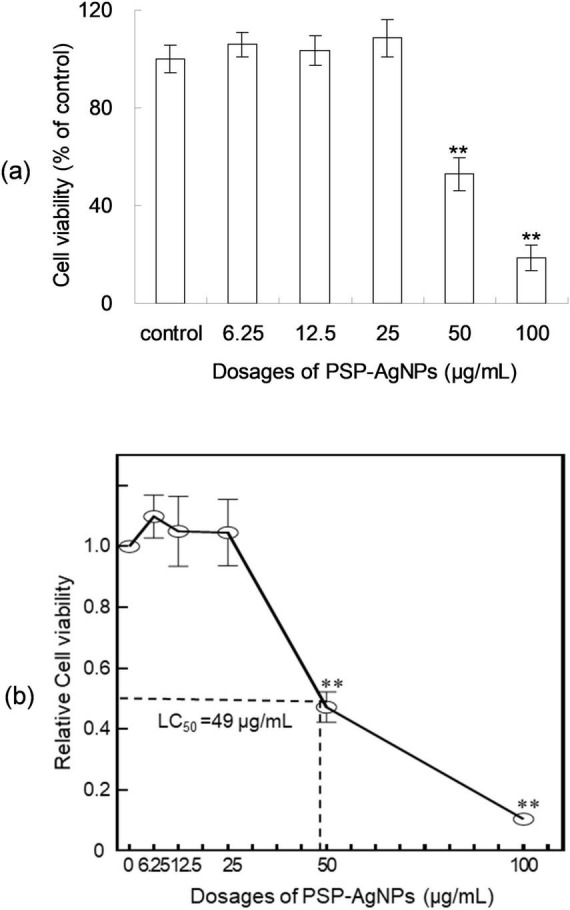
Viability **(a)** and dose-response curve **(b)** of LO2 cells treated with polysaccharide-protein complex silver nanoparticles (PSP-AgNPs) (6.25–100.0 μg/mL) for 24 h. Data are mean ± SD, **P < 0.01 vs. Control.

As shown in Figure 1, PSP-AgNPs showed no cytotoxicity on LO2 cells within the effective dosages of PSP-AgNPs against bacteria (6.25–25.0 μg/mL). However, significantly decreased cellular viability was observed at dosages of 50.0 and 100.0 μg/mL (*p* < 0.01). Compared to control, the cellular viabilities at dosages of 50.0 and 100.0 μg/mL were 43.4 ± 8.59% and 11.38 ± 2.01%, respectively. The dose-response curve of LO2 cells is displayed in Figure 1. The exact concentration of LC_50_ was 49.0 μg/mL.

### 2.3 Intracellular ROS levels and MDA content

**FIGURE 2:**
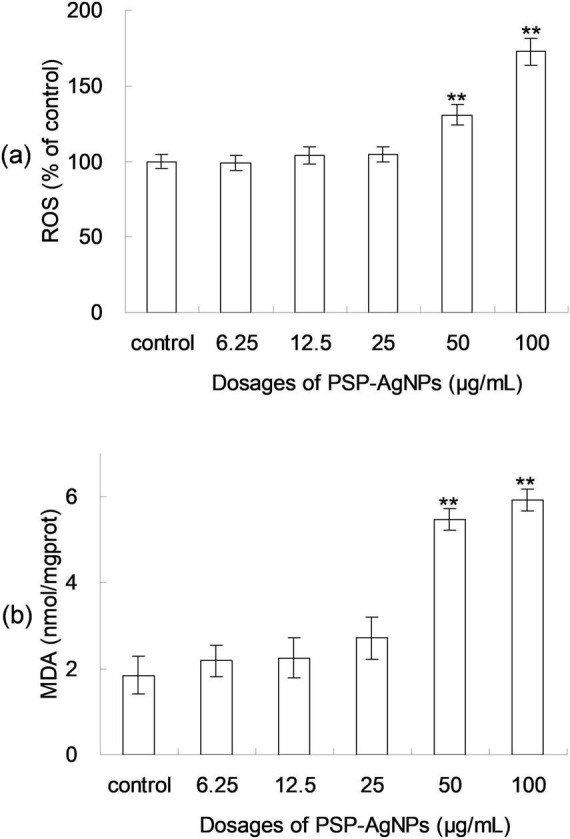
Levels of reactive oxygen species (ROS) **(a)** and malondialdehyde (MDA) content **(b)** in LO2 cells treated with PSP-AgNPs (6.25– 100.0 μg/mL) for 24 h. Data are mean ± SD, **P < 0.01 vs. Control.

Excessive production of ROS induces cellular apoptosis; therefore, the ROS formation after 24 h of PSP-AgNPs exposure was assessed using the 2-7-dichlorodiacetate (DCFH-DA) assay. As shown in Figure 2, the ROS levels increased significantly at concentrations of 50.0 or 100.0 μg/mL compared to Control, and no significant changes were found at other concentration levels (6.25–25.0 μg/mL). As shown in Figure 2, a significant increase of the intracellular MDA (*p* < 0.01) was observed in groups treated with PSP-AgNPs at concentrations of 50.0 and 100.0 μg/mL. Simultaneously, compared to Control, the MDA content did not increase in groups treated with PSP-AgNPs at concentrations of 6.25, 12.5, or 25.0 μg/mL.

### 2.4 Level of intracellular LDH

**FIGURE 3:**
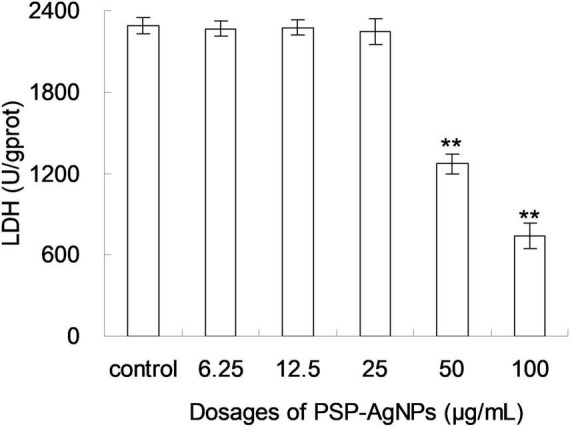
Levels of intracellular lactate dehydrogenase (LDH) in LO2 cells treated with PSP-AgNPs (6.25–100.0 μg/mL) for 24 h. Data are mean ± SD, **P < 0.01 vs. Control.

Based on the above results of cellular viability and MDA content, the level of intracellular LDH in LO2 was further evaluated. As shown in Figure 3, the level of intracellular LDH significantly decreased (*p* < 0.01) in groups exposed to PSP-AgNPs at concentrations of 50.0 and 100.0 μg/mL. However, compared to Control, no significant difference was found in groups treated with PSP-AgNPs at concentrations of 6.25, 12.5, and 25.0 μg/mL.

### 2.5 Activities of antioxidases

**FIGURE 4:**
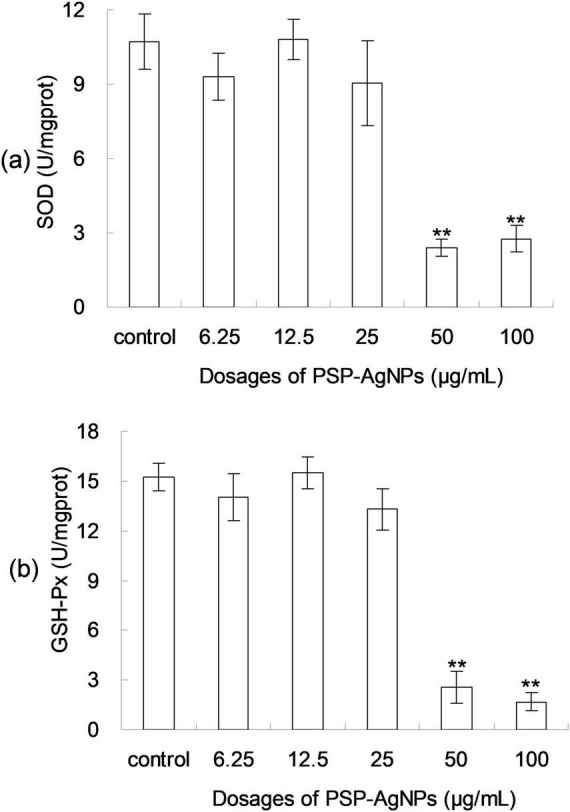
The levels of intracellular superoxide dismutase (SOD) **(a)** and glutathione peroxidase (GSH-Px) **(b)** in LO2 cells treated with PSP-AgNPs (6.25–100.0 μg/mL) for 24 h. Data are mean ± SD, **P < 0.01 vs. Control.

As shown in Figure 4, dose-dependent decreases of SOD and GSH-Px activity were found in LO2 cells exposed to PSP-AgNPs. In the treatment of PSP-AgNPs at concentrations of 50.0 and 100.0 μg/mL, SOD and GSH-Px activity were significantly lower than those in the Control. However, significant differences were not found in other treatments compared to Control. These observations show that the activity of antioxidant enzymes within LO2 were markedly inhibited by PSP-AgNPs exposure at concentrations up to 50.0 μg/mL.

## 3 Discussion

The antibacterial activity of PSP-AgNPs against *V. vulnificus* was superior to that in literature in which a higher dosage of AgNPs, stabilized by carboxy methyl cellulose, was needed (Prema et al., 2017). MIC values and MBC values of AgNPs against *V. vulnificus* were 60.0 and 70.0 μg/mL, respectively. However, the corresponding values of PSP-AgNPs against *V. vulnificus* were 12.50 and 25.0 μg/mL, respectively. A similar phenomenon was also found in AgNPs prepared by the Red alga *Portieria hornemannii*, for which the MIC value against *V. vulnificus* was 15.62 μg/mL (Fatima et al., 2020).

In addition to *V. vulnificus*, PSP-AgNPs also demonstrated superior antibacterial activity against *V. fluvialis* compared to the literature (Meneses-Márquez et al., 2019). Previously, a dosage of 22.5 μg/mL was needed to completely inhibit the growth of *V. fluvialis* by AgNPs, which was prepared by sodium citrate (Meneses-Márquez et al., 2019). However, in the present study, the MIC of PSP-AgNPs against *V. fluvialis* was 12.5 μg/mL. So far, no antibacterial activity of AgNPs against *V. mimicus*, *V. hollisae*, or *V. furnissii* had been reported in the literature. In conclusion, PSP-AgNPs displayed effective antibacterial activity against pathogenic *Vibrio* strains within a concentration range of 3.125–25.0 μg/mL. Additionally, the excellent antibacterial activities demonstrated by PSP-AgNPs should be ascribed to its small average particle size (5 nm) and highly stable dispersion (Jian et al., 2019).

The observations of cellular viability of LO2 cells were in accordance with the results of a previous study, in which PSP-AgNPs were freshly prepared (Jian et al., 2019). This result further confirmed the excellent dispersion stability of PSP-AgNPs as previously reported (Jian et al., 2020). Further, the above findings also showed that PSP-AgNPs injured LO2 cells at dosages exceeding 50.0 μg/mL (*p* < 0.01). This critical toxic concentration of LO2 cells found in PSP-AgNPs approximated to the value reported in the literature, in which LO2 cells maintained normal viability at an exposure level below 80 μg/mL of AgNPs coated with polyvinylpyrrolidone (Jian et al., 2020). Overall, the results of this study confirmed that high dosages of AgNPs exerted cellular damage on LO2 cells, which was also reported in the literature (Piao et al., 2011). Thus, the following determination of oxidative stress were conducted to elucidate the mechanism.

The findings found in our study was partly consistent with the report in the literature, in which AgNPs caused the generation of ROS in a dose-dependent manner in many cell types (Rezvani et al., 2019). However, previous research demonstrated that AgNPs coated by polyvinylpyrrolidone did not induce increased ROS levels in LO2 cells over a concentration range of 20.0–160.0 μg/mL (Xue et al., 2018). This inconsistency between the present study and the literature may be caused by the different physiochemical properties of AgNPs used. This further indicates that the physiochemical properties of AgNPs play an important part in its toxicity and biological effect (Rezvani et al., 2019). The detailed mechanism should be fully examined in the future.

In addition to cell viability and ROS, the content of MDA is also an important index for cellular injuries caused by oxidative stress. Being a byproduct of lipid peroxidation, MDA is a common marker for the quantification of lipid peroxide (Ale et al., 2019). These observations of ROS levels and MDA contents were fully consistent with cellular viability findings. Further, oxidative damage only occurred in LO2 cells when the concentration of PSP-AgNPs exceeded 50.0 μg/mL. In addition, the dose dependent effects of AgNPs on MDA accumulation were also reported before (El Mahdy et al., 2015), in which the tests were done in other cell lines, tissues (El-Samad et al., 2022), or animals (Alwan et al., 2021).

LDH is a soluble yet stable cytoplasmic enzyme that is released into the cell culture medium once the cell membrane is damaged (Alhajjar et al., 2022). Thus, the level of intracellular LDH can be used to assess the integrity of the cell membrane. A decreased level of intracellular LDH indicates an injured cell membrane (Xue et al., 2018). Thus, the observed levels of intracellular LDH demonstrated that the cell membrane was damaged at concentrations of PSP-AgNPs up to 50.0 μg/mL. Cell membranes showed no injury at concentrations of PSP-AgNPs below 25.0 μg/mL. This finding of LDH levels was in accordance with observations of cell viability and MDA content.

Additionally, this toxicity of PSP-AgNPs on cell membrane integrity was lower than that reported in the literature, in which 20.0 μg/mL of AgNPs significantly disrupted the cell membrane integrity of LO2 (Xue et al., 2018). This showed that PSP-AgNPs had lower toxicity compared to other AgNPs. This difference in toxicity may be ascribed to the coating, particle sizes, or preparation methods (Rezvani et al., 2019), and the detailed mechanism should be fully analyzed in the future.

It has been reported that treatment with AgNPs generates elevated intracellular ROS levels and disrupts the activities of antioxidant enzymes (Suliman et al., 2015). SOD and GSH-Px are essential intracellular antioxidant enzymes that help cells to resist oxidative damage. Their ability to remove free radicals is directly proportional to their enzyme activity. Excessive free radicals within cells can trigger cellular toxicity, leading to a reduction in intracellular antioxidant enzyme levels (Nguyen et al., 2020). SOD converts superoxide radical to hydrogen peroxide and oxygen, and thus eliminates cellular damage caused by superoxide radical. Similarly, GSH-Px works on peroxides to prevent cell injury (Fouda et al., 2021).

These findings of antioxidant enzyme activity were contrary to previous research in which AgNPs were found to induce increased level of ROS and SOD, when their concentrations were elevated to a critical value (Suliman et al., 2015; Jiang et al., 2014). The elevated activities of SOD reported in the literature were ascribed to the need to scavenge ROS after exposure to AgNPs (Jiang et al., 2014). An opposing phenomenon in SOD activity was observed between this research and the literature, which may be ascribed to various factor, such as physical-chemical properties of AgNPs, treated subjects, and observation time (Lin et al., 2022). The detailed mechanism should be further studies in the future.

## 4 Materials and methods

### 4.1 Cell culture and materials

The LO2 cell line was obtained from iCell Bioscience Inc. (Shanghai, China). Dulbecco’s modified Eagle medium (DMEM), which is a low glucose liquid medium (cat. no. D6046) was purchased from Merck & Co., Inc. (Rahway, NJ, USA). Fetal bovine serum (cat. no. 10437) was purchased from Invitrogen (Thermo Fisher Scientific, Waltham, MA, USA). Penicillin (cat. no. 87-08-1) and streptomycin (cat. no. 3810-74-0) with purities of up to 99.9% were obtained from Sigma Aldrich (Milwaukee, Missouri, USA). DCFH-DA (cat. no. D6883) was purchased from Merck & Co., Inc.

*Vibrio fluvialis* (ATCC 33809), *Vibrio mimicus* (ATCC 33653), *Vibrio hollisae* (ATCC 35084), *Vibrio vulnificus* (ATCC 27562) and *Vibrio furnissii* (ATCC 35016) were purchased from China General Microbiological Culture Collection Center.

Ultrapure water (18 MΩ, Millipore) was used in all experiments. The assay kits for the determination of MDA (cat. no. A003-4-1), LDH (cat. no. A020-2-2), SOD (cat. no. A001-3-2), and GSH-Px (cat. no. A005-1-2) were supplied by the Nanjing Jian-cheng Bioengineering Institute (Nanjing, China).

### 4.2 PSP and PSP-AgNPs

PSP was obtained in our previous research, via hydrolysis of viscera *of Haliotis discus* upon further purification of membrane filtration and gel permeation chromatography (Jian et al., 2019). The weight-averaged molecular weight (Mw) of PSP was 25.38 ± 0.75 kDa with a polydispersity index of 1.181 ± 1.32. A random coil conformation was found in PSP, with a root-mean-square radius (Rz) of 32.23 ± 2.76 nm. The contents of sugar and protein of PSP were 55.51 ± 0.43% and 27.01 ± 0.54%, respectively. Seven types of monosaccharide were found in the polysaccharides of PSP, and the protein of PSP was composed of 18 types of amino acids. Detailed information about PSP can be found in our previous study (Jian et al., 2019).

Based on the obtained PSP, PSP-AgNPs was prepared using a simple redox system of silver nitrate, using PSP as both a reducing and capping agent. AgNPs were firmly capped by PSP through the formation of Ag-O, Ag-N, and Ag-S bonds. An average particle size of 6.3 ± 2.4 nm, a spherical morphology, and cubic face-centered silver were found in AgNPs. The hydrodynamic diameter, polydispersity index, and zeta potential of PSP-AgNPs were 79.5 ± 10.4 nm, 0.39 ± 0.024, and −33.9 ± 3.6 mV, respectively, when it was dispersed in de-ionized water at pH 7.0. The silver content in PSP-AgNPs was approximately 10.10 ± 0.54% (w/w), as detected by inductively coupled plasma optical emission spectroscopy. Other physiochemical properties and preparation of PSP-AgNPs are fully described in our previous studies (Jian et al., 2020; Jian et al., 2019).

### 4.3 Preparation of PSP-AgNPs dispersion

The dispersion of PSP-AgNPs was prepared by dispersing the lyophilized powder of PSP-AgNPs into ultrapure water at a concentration of 1.0 mg/mL. The lyophilized powder of PSP-AgNPs had been stored for 6 months in a desiccator at room temperature. Then, the dispersion of PSP-AgNPs was stored at 4°C for further use.

### 4.4 Antibacterial assays against pathogenic *Vibrio* strains

Antibacterial assays on the cultures of pathogenic *Vibrio* strains (*V. fluvialis*, *V. mimicus*, *V. hollisae*, *V. vulnificus*, and *V. furnissii*) were conducted using the procedure of broth micro-dilution (Jian et al., 2020). Mueller-Hinton broth medium was purchased from Guangdong Huankai Bio-Technology Co., Ltd., Guangzhou, China. This medium was composed of 5 g/l glucose, 10 g/l beef extract, 10 g/l peptone, 3 g/l yeast extract, 1 g/l soluble starch, 0.5 g/l cysteine HCl, 5 g/l sodium chloride, 3 g/l sodium acetate, and 0.5 g/l agar.

*Vibrio* inoculum was prepared in advance, adjusted to a concentration of 1 × 10^8^ CFU/mL using a Densimat, and then diluted to 1 × 10^6^ CFU/mL using Mueller-Hinton broth medium. After dilution, 1 mL of bacterial suspensions and 1 mL of serial dilutions of PSP-AgNPs (200.0, 100.0, 50.0, 25.0, 12.5, 6.25, 3.125, or 1.56 μg/mL) dispersed in Mueller-Hinton broth were blended in treatment tubes with a capacity of 10 mL. The total volume of fermentation broth in each treated tube was 2 mL. The treatment tubes were incubated at 37°C for 24 h under aerobic conditions. After cultivation, MIC was defined through treatment tubes without bacterial growth in the highest dilution of PSP-AgNPs. Based on the results of MIC, MBC was determined by measurements of bacterial colonies on agar plates after incubation at 37°C for 48 h, using aliquots from treatment tubes without bacterial growth. Briefly, 100.0 μl of these aliquots were withdrawn from treatment tubes showing no visible growth and were spread on agar plates containing Mueller-Hinton broth medium. These plates were then incubated at 37°C for 48 h under aerobic conditions. After this incubation period, the highest dilution which inhibited colony formation on agar was noted as MBC. Each assay was done in quintuples.

### 4.5 Cell culture and assay on cytotoxicity

Dispersions of PSP-AgNPs (1.0 mg/mL) were prepared in DMEM without fetal bovine serum, and further diluted to the required concentration using DMEM before cell cultivation. To ensure homogeneity, the final dispersion of PSP-AgNPs was vortexed vigorously for 1 min and sonicated for 3 min.

Freshly prepared DMEM containing 10% fetal bovine serum, penicillin (100.0 U/mL), and streptomycin (100.0 μg/mL) was used to culture LO2 cells. The cultivation utilized 96-well plates under a humidified atmosphere of 95% air and 5% CO_2_ at 37°C (Jian et al., 2017). After cultivation for three passages, LO2 cells (5 × 10^4^ mL^–1^) were grown in medium containing PSP-AgNPs, the concentration of which was set to 0 (blank control), 6.25, 12.5, 25.0, 50.0, or 100.0 μg/mL. After cultivation for 24 h in 96-well plates under a humidified atmosphere of 95% air and 5% CO_2_ at 37°C, the supernatant was discarded, and cells were washed twice with phosphate buffer solution. Then, 200 μl MTT (0.5 mg/ml) was added, and the mixture was further incubated for 4 h. Thereafter, the supernatant was removed. Finally, 20 ul of dimethyl sulfoxide was added and the mixture was shaken for 2 min using a vortex mixer, followed by measuring the optical density at 490 nm using a microplate reader (Bio-Rad Laboratories, Hercules, CA, USA). Viability was calculated as the ratio of the mean of optical density obtained for each condition to that of the control (Jian et al., 2019).

### 4.6 Determination of the oxidative stress response of LO2 cells

After incubation and treatment with PSP-AgNPs as mentioned in Section 4.5, the medium was discarded, and cells were collected for the measurement of intracellular LDH, MDA, SOD, and GSH-Px by the following protocols. The cells were homogenized via sonication at 300 W for 1 min after scraping into ice-cold phosphate buffer solution. Then, the homogenate was centrifuged (12000 × g, 30 min, 4°C) and the supernatant was collected for determination via LDH assay kit (cat. no. A020-2-2), MDA assay kit (cat. no. A003-4-1), SOD assay kit (cat. no. A001-3-2), or GSH-Px assay kit (cat. no. A005-1-2), respectively.

### 4.7 Measurement of ROS

Based on the literature, the intracellular ROS levels were measured by the DCFH-DA method (Huang et al., 2021). After separation as mentioned in Section 4.5, LO2 cells were further incubated for 30 min in the dark with DMEM containing DCFH-DA (10 mM) under a humidified atmosphere of 95% air and 5% CO_2_ at 37°C in 96-well plates. Thereafter, the medium was discarded. Cells were washed three times with 200 μL of PBS and fixed with 4% paraformaldehyde for 10 min. Afterwards, the treated cells were used to measure the fluorescence intensity using a safire fluorescence plate reader, at an excitation wavelength of 488 nm and an emission wavelength of 525 nm. Finally, values are expressed as percentages of fluorescence intensity relative to control.

### 4.8 Statistical analysis

All experiments were performed in quintuple. SPSS 16.0 software was used to conduct analyses of variance with Student’s *t* test (*P* < 0.01). The results are expressed as means ± standard deviations. The significance level of *P* < 0.01 is labeled with double asterisks in all figures.

## 5 Conclusion

PSP-AgNPs showed no cytotoxicity on LO2 cells within effective dosage ranges against pathogenic *Vibrio* bacteria (3.125–25.0 μg/mL), and serious cytotoxicity was observed when the concentration was increased up to 50.0 μg/mL. Intracellular oxidative stress was the predominant mechanism of toxicity PSP-AgNPs induced in LO2 cells. Overall, this study showed that PSP-AgNPs are highly biocompatible in the range of effective antibacterial dosages; therefore, PSP-AgNPs can be used as a potential bactericide against pathogenic *Vibrio* strains, because of their suitable dispersion behavior, antibacterial activity, and biosafety.

## Data Availability

The original contributions presented in the study are included in the article/supplementary material, further inquiries can be directed to the corresponding author.

## References

[B1] Abioye O. Okoh A. (2018). Limpet (Scutellastra cochlear) recovered from some estuaries in the Eastern Cape Province, South Africa act as reservoirs of pathogenic Vibrio species. *Front. Public Health* 6:381997. 10.3389/fpubh.2018.00237 30234084 PMC6128111

[B2] Ale A. Liberatori G. Vannuccini M. Bergami E. Ancora S. Mariotti G. (2019). Exposure to a nanosilver-enabled consumer product results in similar accumulation and toxicity of silver nanoparticles in the marine mussel Mytilus galloprovincialis. *Aquat. Toxicol.* 211 46–56. 10.1016/j.aquatox.2019.03.018 30946994

[B3] Alhajjar R. Roche K. Techtmann S. (2022). Comparative Analysis of the Mechanism of Resistance to Silver Nanoparticles and the Biocide 2, 2-Dibromo-3-Nitrilopropionamide. *Antimicrob. Agents Chemother.* 66 e2031–e2021. 10.1128/aac.02031-21 35604211 PMC9211392

[B4] Alwan S. Al-Saeed M. Abid H. (2021). Safety assessment and biochemical evaluation of the effect of biogenic silver nanoparticles (using bark extract of C. zeylanicum) on Rattus norvegicus rats. *Baghdad J. Biochem. Appl. Biol. Sci.* 2 133–145. 10.47419/bjbabs.v2i03.67

[B5] Brumfield K. Usmani M. Chen K. Gangwar M. Jutla A. Huq A. (2021). Environmental parameters associated with incidence and transmission of pathogenic Vibrio spp. *Environ. Microbiol.* 23 7314–7340. 10.1111/1462-2920.15716 34390611

[B6] Chandrakala N. Parameswari P. (2021). Comparative study on the in vitro antibacterial activity of selected medicinal plants against pathogenic vibrio species from diseased penaeus monodon (fab). *J. Adv. Sci. Res.* 12(04 Suppl. 1), 288–291. 10.55218/JASR.s1202112433

[B7] El Mahdy M. Eldin T. Aly H. Mohammed F. Shaalan M. (2015). Evaluation of hepatotoxic and genotoxic potential of silver nanoparticles in albino rats. *Exp. Toxicol. Pathol.* 67 21–29. 10.1016/j.etp.2014.09.005 25446800

[B8] El-Samad L. Bakr N. El-Ashram S. Radwan E. Aziz K. Hussein H. (2022). Silver nanoparticles instigate physiological, genotoxicity, and ultrastructural anomalies in midgut tissues of beetles. *Chemico-Biol. Interact.* 367 110166. 10.1016/j.cbi.2022.110166 36087814

[B9] Fatima R. Priya M. Indurthi L. Radhakrishnan V. Sudhakaran R. (2020). Biosynthesis of silver nanoparticles using red algae Portieria hornemannii and its antibacterial activity against fish pathogens. *Microb. Pathog.* 138 103780. 10.1016/j.micpath.2019.103780 31622663

[B10] Fleischmann S. Herrig I. Wesp J. Stiedl J. Reifferscheid G. Strauch E. (2022). Prevalence and distribution of potentially human pathogenic Vibrio spp. on German North and Baltic Sea coasts. *Front. Cell. Infect. Microbiol.* 12:846819. 10.3389/fcimb.2022.846819 35937704 PMC9355094

[B11] Fouda M. Dosoky W. Radwan N. Abdelsalam N. Taha A. Khafaga A. (2021). Oral administration of silver nanoparticles–adorned starch as a growth promotor in poultry: Immunological and histopathological study. *Int. J. Biol. Macromol.* 187 830–839. 10.1016/j.ijbiomac.2021.07.157 34331979

[B12] Huang M. Ye K. Hu T. Liu K. You M. Wang L. (2021). Silver nanoparticles attenuate the antimicrobial activity of the innate immune system by inhibiting neutrophil-mediated phagocytosis and reactive oxygen species production. *Int. J. Nanomed.* 2021 1345–1360. 10.2147/IJN.S292482 33633450 PMC7901559

[B13] Jian W. Ma Y. Wu H. Zhu X. Wang J. Xiong H. (2019). Fabrication of highly stable silver nanoparticles using polysaccharide-protein complexes from abalone viscera and antibacterial activity evaluation. *Int. J. Biol. Macromol.* 128 839–847. 10.1016/j.ijbiomac.2019.01.197 30710585

[B14] Jian W. Ma Y. Zhu X. Zhang N. Lin L. Jia B. (2020). Quantitative insight into dispersity and antibactericidal capability of silver nanoparticles noncovalently conjugated by polysaccharide-protein complexes. *Int. J. Biol. Macromol.* 150 459–467. 10.1016/j.ijbiomac.2020.02.098 32057866

[B15] Jian W. Tu L. Wu L. Xiong H. Pang J. Sun Y. (2017). Physicochemical properties and cellular protection against oxidation of degraded Konjac Glucomannan prepared by γ-irradiation. *Food Chem.* 231 42–50. 10.1016/j.foodchem.2017.03.121 28450022

[B16] Jiang H. Qiu X. Li G. Li W. Yin L. (2014). Silver nanoparticles induced accumulation of reactive oxygen species and alteration of antioxidant systems in the aquatic plant Spirodela polyrhiza. *Environ. Toxicol. Chem.* 33 1398–1405. 10.1002/etc.2577 24619507

[B17] Komazec B. Cvjetko P. Balen B. Letofsky-Papst I. Lyons D. Peharec Stefaniæ P. (2023). The occurrence of oxidative stress Induced by Silver nanoparticles in Chlorella vulgaris depends on the surface-stabilizing Agent. *Nanomaterials* 13 1967. 10.3390/nano13131967 37446486 PMC10343332

[B18] Lin X. Lin Y. Liao Z. Niu X. Wu Y. Shao D. (2022). Preservation of Litchi Fruit with Nanosilver Composite Particles (Ag-NP) and Resistance against Peronophythora litchi. *Foods* 11 2934. 10.3390/foods11192934 36230009 PMC9564286

[B19] Lu C. Lv Y. Kou G. Liu Y. Liu Y. Chen Y. (2022). Silver nanoparticles induce developmental toxicity via oxidative stress and mitochondrial dysfunction in zebrafish (Danio rerio). *Ecotoxicol. Environ. Saf.* 243 113993. 10.1016/j.ecoenv.2022.113993 35994909

[B20] Meneses-Márquez J. Hamdan-Partida A. Del Carmen Monroy-Dosta M. Castro-Mejía J. Faustino-Vega A. Soria-Castro E. (2019). Use of silver nanoparticles to control Vibrio fluvialis in cultured angelfish Pterophyllum scalare. *Dis. Aquatic Organ.* 137 65–72. 10.3354/dao03423 31802743

[B21] Neetoo H. Reega K. Manoga Z. Nazurally N. Bhoyroo V. Allam M. (2022). Prevalence, genomic characterization, and risk assessment of human pathogenic Vibrio Species in Seafood. *J. Food Prot.* 85 1553–1565. 10.4315/JFP-22-064 35880931

[B22] Nguyen N. Tran G. Nguyen C. (2020). Anti-oxidative effects of superoxide dismutase 3 on inflammatory diseases. *J. Mol. Med.* 98 59–69. 10.1007/s00109-019-01845-2 31724066

[B23] Piao M. Kang K. Lee I. Kim H. Kim S. Choi J. (2011). Silver nanoparticles induce oxidative cell damage in human liver cells through inhibition of reduced glutathione and induction of mitochondria-involved apoptosis. *Toxicol. Lett.* 201 92–100. 10.1016/j.toxlet.2010.12.010 21182908

[B24] Prema P. Thangapandiyan S. Immanuel G. (2017). CMC stabilized nano silver synthesis, characterization and its antibacterial and synergistic effect with broad spectrum antibiotics. *Carbohydrate Polym.* 158 141–148. 10.1016/j.carbpol.2016.11.083 28024537

[B25] RathnaKumari P. Kolanchinathan P. Siva D. Abirami B. Masilamani V. John G. (2018). Antibacterial efficacy of seagrass Cymodocea serrulata-engineered silver nanoparticles against prawn pathogen Vibrio parahaemolyticus and its combative effect on the marine shrimp Penaeus monodon. *Aquaculture* 493 158–164. 10.1016/j.aquaculture.2018.04.061

[B26] Rezvani E. Rafferty A. McGuinness C. Kennedy J. (2019). Adverse effects of nanosilver on human health and the environment. *Acta Biomater.* 94 145–159. 10.1016/j.actbio.2019.05.042 31125729

[B27] Salama B. Alzahrani K. Alghamdi K. Al-Amer O. Hassan K. Elhefny M. (2023). Silver nanoparticles enhance oxidative stress, inflammation, and apoptosis in liver and kidney tissues: Potential protective role of thymoquinone. *Biol. Trace Element Res.* 201 2942–2954. 10.1007/s12011-022-03399-w 36018545

[B28] Serrano-Díaz P. Williams D. Vega-Arreguin J. Manisekaran R. Twigg J. Morse D. (2023). Geranium leaf-mediated synthesis of silver nanoparticles and their transcriptomic effects on Candida albicans. *Green Process. Synth.* 12 20228105. 10.1515/gps-2022-8105

[B29] Sony M. Sumithra T. Anusree V. Amala P. Reshma K. Alex S. (2021). Antimicrobial resistance and virulence characteristics of Vibrio vulnificus, Vibrio parahaemolyticus and Vibrio harveyi from natural disease outbreaks of marine/estuarine fishes. *Aquaculture* 539 736608. 10.1016/j.aquaculture.2021.736608

[B30] Suliman Y. A. Ali D. Alarifi S. Harrath A. Mansour L. Alwasel S. (2015). Evaluation of cytotoxic, oxidative stress, proinflammatory and genotoxic effect of silver nanoparticles in human lung epithelial cells. *Environ. Toxicol.* 30 149–160. 10.1002/tox.21880 23804405

[B31] Xue Y. Wang J. Huang Y. Gao X. Kong L. Zhang T. (2018). Comparative cytotoxicity and apoptotic pathways induced by nanosilver in human liver HepG2 and L02 cells. *Hum. Exper. Toxicol.* 37 1293–1309. 10.1177/0960327118769718 29658330

[B32] Xue Y. Zhang T. Zhang B. Gong F. Huang Y. Tang M. (2016). Cytotoxicity and apoptosis induced by silver nanoparticles in human liver HepG2 cells in different dispersion media. *J. Appl. Toxicol.* 36 352–360. 10.1002/jat.3199 26198703

[B33] Yang X. Wu J. (2022). Synthetic Conditions, Physical Properties, and Antibacterial Activities of Silver Nanoparticles with Exopolysaccharides of a Medicinal Fungus. *Materials* 15 5620. 10.3390/ma15165620 36013754 PMC9412466

